# Associated factors with poor treatment response to initial glucocorticoid therapy in patients with adult-onset Still’s disease

**DOI:** 10.1186/s13075-022-02780-3

**Published:** 2022-04-29

**Authors:** Fumiaki Kondo, Takahiko Sugihara, Natsuka Umezawa, Hisanori Hasegawa, Tadashi Hosoya, Naoki Kimura, Masaaki Mori, Shinsuke Yasuda

**Affiliations:** 1 Department of Lifetime Clinical Immunology, Graduate School of Medical and Dental Science, Tokyo Medical and Dental University (TMDU), Tokyo, Japan; 2 Department of Rheumatology, Graduate School of Medical and Dental Science, Tokyo Medical and Dental University (TMDU), Tokyo, Japan; 3 Current address: Division of Rheumatology and Allergy, Department of Internal Medicine, St. Marianna University School of Medicine, 2-16-1, Sugao, Miyamae-ku, Kawasaki, 216-8511 Japan

**Keywords:** Adult-onset Still’s disease, Poor prognostic factor, White blood cell

## Abstract

**Background:**

High-dose glucocorticoids (GC) are first-line treatment for adult-onset Still’s disease (AOSD); however, some of the patients remain refractory to initial GC therapy, or rapidly relapse. The aim of this study was to identify prognostic factors for poor treatment response to initial GC therapy for AOSD.

**Methods:**

Data on newly diagnosed AOSD patients were extracted from our database (*n*=71, mean age 51.6 years). The primary outcome was a poor treatment outcome at 4 weeks, which was defined as failure to achieve remission or relapse after achieving remission within 4 weeks, followed by administration of two or more rounds of GC pulse therapy or of any other immunosuppressive drugs.

**Results:**

The initial mean dose ± standard deviation of prednisolone was 0.82 ± 0.23 mg/kg/day, and 34 (47.3%) patients received GC pulse therapy at week 0. Twenty-nine of 71 patients exhibited a poor treatment outcome at 4 weeks (40.8%). The second round of GC pulse therapy or immunosuppressive drugs was added in 17 or 24 of the 29 patients, respectively. These patients had higher baseline white blood cell (WBC) counts, serum ferritin levels, systemic feature score based on clinical symptoms (modified systemic feature score, mSFS), more hemophagocytic syndrome (HPS) over the 4 weeks, and the higher severity score based on modified Pouchot score or severity index of the Japanese Ministry of Health, Labour and Welfare, than the remaining 42 patients. Multivariable logistic regression model identified baseline WBC count as a prognostic factor for poor outcome (odds ratio per 1000/μl increment: 1.12, 95% CI 1.04–1.29), while thrombocytopenia, hyperferritinemia, and mSFS at baseline did not achieve statistical significance. Receiver-operating characteristic curve analysis showed that the optimal cut-off for WBC count was 13,050/μl. The Kaplan-Meier method showed the cumulative rate of poor treatment outcome to be 60.0% in patients with WBC ≥13,050/μl and 23.5% in those with WBC <13,050/μl.

**Conclusions:**

A higher WBC count but not thrombocytopenia, hyperferritinemia, and mSFS at baseline was a significant prognostic factor for poor treatment outcome at week 4 in this retrospective cohort of AOSD patients. Our findings provide important information for determining the initial treatment strategy of newly-diagnosed AOSD.

**Supplementary Information:**

The online version contains supplementary material available at 10.1186/s13075-022-02780-3.

## Background

Adult-onset Still’s disease (AOSD) is a systemic inflammatory disease first reported by Bywaters in 1971 [1]. Clinical signs and symptoms of AOSD include fever, arthritis, typical skin rash, myalgia, lymphadenopathy and serositis, and hemophagocytic syndrome (HPS) [2]. While high-dose glucocorticoids (GC) are first-line treatment for AOSD [3], GC pulse therapy is frequently added to oral GC in the induction treatment regimen [4]. However, some patients remain refractory to the induction therapy, or rapid relapse; hence, there is an unmet need to understand treatment failures and develop alternative strategies.

A recent randomized controlled trial has documented the efficacy of the anti-IL-6 receptor antibody tocilizumab for AOSD refractory to GC therapy [5]. As we embark on a new era of AOSD treatment and develop novel treatment strategies, prognostic factors for initial treatment response to standard doses of oral GC with or without GC pulse therapy are important for determining indications for additional treatments including other immunosuppressive drugs or tocilizumab.

Severe macrophage activation syndrome (MAS) often accompanies a fatal course of systemic juvenile idiopathic arthritis (sJIA), associated with multiple organ damage, disseminated intravascular coagulation (DIC), and HPS [6]. Because the clinical features of AOSD are similar to sJIA [7], and severe MAS in both sJIA or AOSD is treated with repeated GC pulse therapy together with high-dose oral GC, immunosuppressive drugs, TNF inhibitors, or tocilizumab [8–10], the experience with sJIA suggests that high serum ferritin and cytopenia due to MAS might be associated with poor treatment response to initial GC therapy also in AOSD. Thus, we hypothesized that white blood cell (WBC) counts, platelet (Plt) counts, aspartate aminotransferase (AST), lactate dehydrogenase (LDH), ferritin, and clinical signs and symptoms of organ damage due to AOSD determine the patient’s response to initial high-dose GC. Here, we have investigated whether these candidate factors are associated with poor treatment outcomes in patients with AOSD using 1-year follow-up data from our retrospective cohort study.

## Methods

### Database

Patients aged ≥16 years with a diagnosis of AOSD were selected consecutively from 2007 to 2019 from the inpatient database of Tokyo Medical and Dental University. The inpatient database was created for all hospitalized patients, and data on all of those with AOSD were extracted without any exclusions. Patient data were extracted using a pre-defined case report form at 1 week and 2, 3, 4, 8, and 52 weeks after the start of GC treatment. The diagnosis of AOSD was made at the discretion of the attending physician based on Yamaguchi’s classification criteria [11]. Clinical signs and symptoms of AOSD, WBC, neutrophil-lymphocyte ratio (NLR), Plt, AST, LDH, and serum ferritin at each time point were extracted from the medical records.

### Outcomes

The primary outcome was a poor treatment outcome at 4 weeks, i.e., having a poor treatment response event defined as administration of two or more rounds of GC pulse therapy or of any other immunosuppressive drugs within 4 weeks due to failure to achieve remission or due to relapse after achieving remission. Remission was indicated by the resolution of clinical signs and symptoms related to AOSD activity (defined as having fever (>39.0°C), weight loss or fatigue, myalgia, arthritis, rashes, lymphadenopathy, sore throat, hepatosplenomegaly, abnormal liver function tests, pericarditis, pleuritis, interstitial lung disease, HPS, DIC, and/or elevation of C-reactive protein (CRP) levels). Weight loss was defined as a decrease of 1kg or more. Arthritis was defined as swelling and tenderness confirmed by a rheumatologist. Rash was considered to be present if patients had a salmon-pink rash predominantly on the trunk and extremities, confirmed by rheumatologist or dermatologist. Hepatomegaly and splenomegaly were confirmed by ultrasonography (US) or computed tomography (CT). Lymphadenopathy was defined as the presence of enlarged lymph nodes over 1 cm. Pericarditis was defined as abnormal pericardial effusion documented by echocardiography or CT at diagnosis of AOSD. Pleuritis was defined as the exudative pleural effusion at diagnosis of AOSD or the pleural effusion with pleuritic pain documented by CT. Interstitial pneumonia was confirmed by chest CT. An elevated CRP level was defined as > 0.3 mg/dl. The presence of HPS was determined based on definitive diagnosis by bone marrow aspiration or biopsy. The diagnosis of DIC was made based on the diagnostic criteria of the Japanese Society on Thrombosis and Hemostasis [12]. Relapse was determined as having occurred if the AOSD activity reappeared after achievement of clinical remission, and required increased GC doses or the addition of immunosuppressive drugs. Elevated CRP alone without clinical signs and symptoms was not considered to be relapse.

### Definition of the modified systemic feature score, modified Pouchot score, and severity index

The severity of organ damage due to AOSD was evaluated using items describing clinical signs and symptoms in the original systemic feature score [13] (modified systemic feature score, mSFS). This included fever, rash, lymphadenopathy, hepatosplenomegaly, and serositis. Each clinical feature was assigned a score of 1 (present) or 0 (absent).

Modified Pouchot scores [14] and the severity index of the Japanese Ministry of Health, Labour and Welfare (Severity Index) [15] were also evaluated. The modified Pouchot score included fever, evanescent rashes, sore throat, arthritis, myalgia, pleuritis, pericarditis, pneumonitis, lymphadenopathy, hepatomegaly or abnormal liver function tests, elevated leukocyte counts (>15,000/μl), and serum ferritin (>3000 μg/l). Each clinical or laboratory feature was also assigned a score of 1 (present) or 0 (absent). The Severity Index was calculated as the sum of the following scores: serositis (1), neutrophil ratio >85% (1), serum ferritin >3000 ng/ml (1), prominent lymphadenopathy (1), refractoriness to GC therapy (>0.4 mg/kg of prednisolone equivalent) (1), HPS (2), and DIC (2).

### Safety

Serious adverse events of interest were assessed by collecting information for 0–52 weeks after the start of treatment. The development of serious infections including bacterial pneumonia, other bacterial infections, pneumocystis pneumonia, deep fungal infections, herpes zoster, tuberculosis, non-tuberculous mycobacterial infections and cytomegalovirus infection, fractures, and death were evaluated.

### Statistics

Student’s *t* test and the Mann-Whitney test were used to compare continuous variables depending on their distribution, and the chi-square test and Fisher’s exact test were used for categorical variables. The correlation coefficients were evaluated using Pearson’s product moment correlation coefficient or Spearman’s rank correlation coefficient.

Univariable analysis and multivariable analysis for factors associated with poor treatment outcomes at 4 weeks were conducted using logistic regression analysis. Receiver-operating characteristic (ROC) curves were constructed to evaluate the predictivity of identifying patients with poor treatment outcomes. Cumulative rates and median time to the first event of the poor treatment outcomes within 28 days were analyzed using the Kaplan-Meier method and log-rank testing. All statistical analyses were performed using IBM Statistical Package for the Social Sciences version 24 (IBM, Armonk, NY, USA). All reported *p* values are two-tailed, and the level of significance is taken as *p* < 0.05.

## Results

### Patient characteristics

The case report forms of 71 newly diagnosed Japanese AOSD patients were assessed, confirming that all of them satisfied Yamaguchi’s classification criteria after exclusion of those with infectious, neoplastic, and other autoimmune disorders. The mean age ± standard deviation (S.D.) was 51.6 ± 18.1, 50 were women (70.4%), 42 (59.2%) had high fever (>39°C) at baseline, 55 (77.5%) had a typical rash, 50 (70.4%) had a sore throat, 47 (66.2%) had arthritis, and 27 (14.1%) had HPS (Table 1).

**Table 1:** Clinical characteristics of patients at onset

	All (*n*=71)	With the event^a^ (*n*=29)	Without the event (*n*=42)	*p*-value
Age, years, mean ± S.D.	51.6 ± 18.1	54.5 ± 18.9	49.6 ± 17.5	0.262
Female patients, %	70.4	69.0	71.4	0.823
Weight, kg, mean ± S.D.	56.6 ± 12.1	56.5 ± 9.55	56.6 ± 13.8	0.967
Fever (>39.0°C), %	59.2	72.4	50.0	0.059
Weight loss or fatigue, %	62.0	55.2	66.7	0.327
Myalgia, %	32.4	37.9	28.6	0.407
Rashes, %	77.5	79.3	76.2	0.757
Lymphadenopathy, %	71.8	75.9	69.0	0.530
Sore throat, %	70.4	75.9	66.7	0.404
Hepatosplenomegaly, %	60.6	69.0	54.8	0.229
Pericarditis, %	7.0	13.8	2.4	0.086
Serositis, %	12.7	27.6	2.4	0.003
Interstitial lung disease, %	7.0	10.3	4.8	0.327
Arthritis, %	66.2	72.4	63.4	0.430
Hemophagocytic syndrome, %	14.1	24.1	7.1	0.048
DIC, %	5.6	6.9	4.8	0.542
mSFS score, mean ± S.D.	3.25 ± 0.91	3.59 ± 0.91	3.02 ± 0.84	0.009
WBC, /μl, mean ± S.D.	13919 ± 7018	17097 ± 7087	11615 ± 6072	0.001
neutrophil, /μl, mean ± S.D.	11871 ± 6734	14855 ± 6832	9651 ± 5806	0.001
lymphocyte, /μl, mean ± S.D.	1033 ± 609	1022 ± 705	1042 ± 534	0.893
NLR, mean ± S.D.	15.27 ± 11.89	20.14 ± 13.72	11.56 ± 8.79	0.005
Hb, g/dl, mean ± S.D.	11.1 ± 1.7	11.2 ± 1.4	11.1 ± 1.9	0.866
Plt, ×10^4^/μl, mean ± S.D.	27.2 ± 13.6	24.6 ± 11.6	29.1 ± 14.8	0.178
ESR, mean ± S.D.	84.5 ± 35.3	75.1 ± 35.2	90.2 ± 34.6	0.652
CRP, mg/dl, mean ± S.D.	12.7 ± 8.9	14.7 ± 8.8	11.2 ± 8.8	0.102
AST, U/l, median (interquartile range)	53.5 (30.0–85.0)	69.0 (51.0–137.0)	45.0 (23.0–82.0)	0.011
ALT, U/l, median (interquartile range)	43.3 (20.0–81.0)	47.5 (27.0–109.0)	34.0 (17.0–70.0)	0.081
LDH, U/l, median (interquartile range)	438 (230–670)	612 (390–928)	331 (198–547)	<0.001
Ferritin, ng/ml, median (interquartile range)	4548 (616–10,323)	5850 (3459–32,902)	1337 (428–7898)	0.001
IgG, mg/dl, mean ± S.D.	1551 ± 747	1372 ± 438	1687 ± 896	0.093
Modified Pouchot score, mean ± S.D.	6.25 ± 1.8	7.28 ± 1.62	5.55 ± 1.57	<0.001
Severity index, mean ± S.D.	3.32 ± 1.62	4.14 ± 1.64	2.76 ± 1.36	<0.001
Calendar year of the onset	2012.5 ± 4.1	2012.8 ± 4.3	2012.3 ± 4.1	0.745

*S.D.* standard deviation, *DIC* disseminated intravascular coagulation, *mSFS* modified systemic feature score, *WBC* white blood cell, *NLR* neutrophil-lymphocyte ratio, *Hb* hemoglobin, *Plt* platelet count, *ESR* erythrocyte sedimentation rate, *CRP* C-reactive protein, *LDH* lactate dehydrogenase, *AST* aspartate transferase, *ALT* alanine aminotransferase, *Severity index* severity index of Japanese Ministry of Health, Labour and Welfare

^a^Event of a poor treatment outcome, which was defined as failure to achieve remission or relapse after achieving remission within 4 weeks, followed by administration of two or more rounds of GC pulse therapy or of any other immunosuppressive drugs

### Initial treatment response to GC therapy

**Fig. 1:**
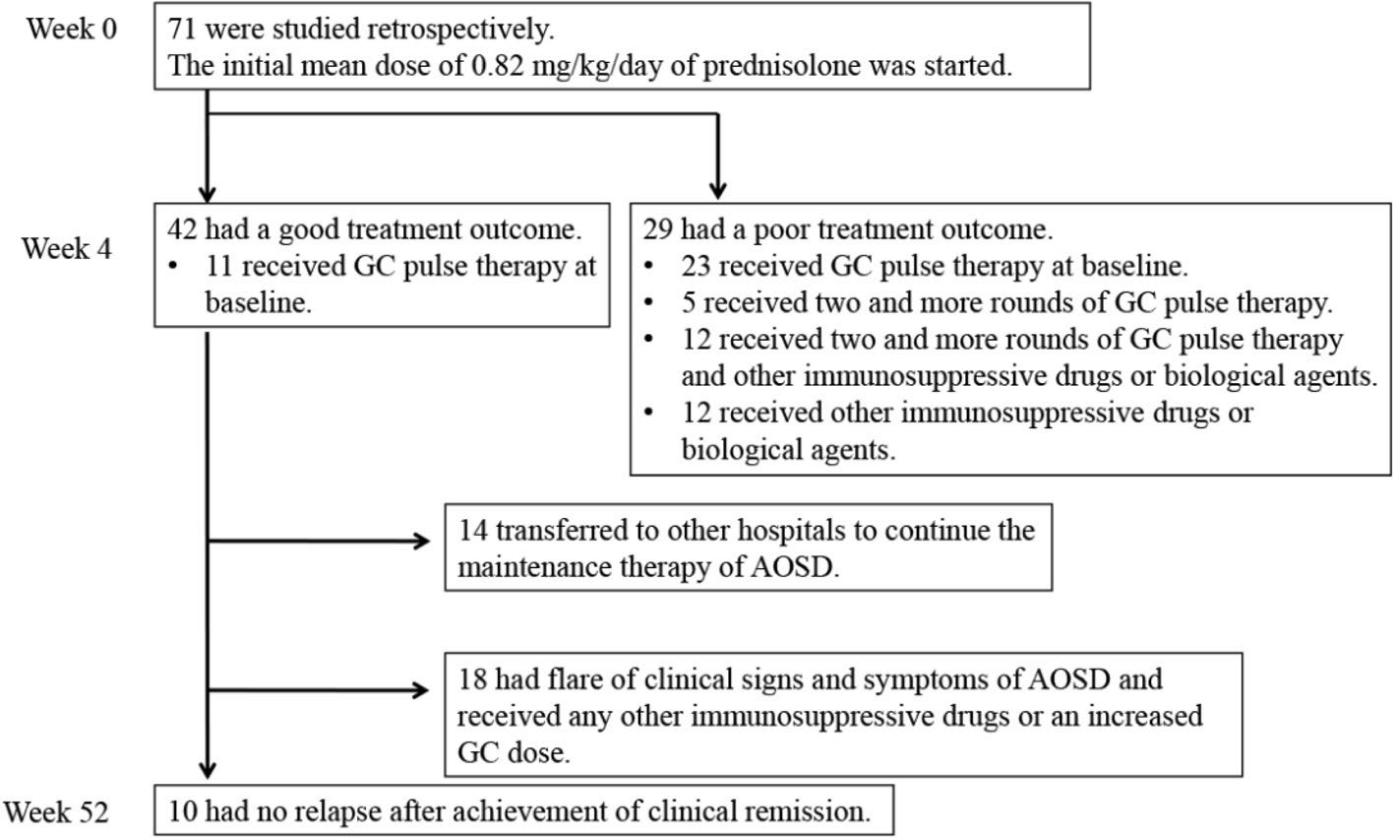
Patient screening and follow-up, and treatment outcomes. The initial mean ± S.D. dose of 0.82 ± 0.23 mg/kg/day of prednisolone was started in 71 newly diagnosed patients, with 34 also receiving glucocorticoid (GC) pulse therapy at baseline. A poor treatment outcome was defined as a poor treatment response within 4 weeks. The latter was defined as administration of two or more rounds of GC pulse therapy or the addition of any other immunosuppressive drugs within 4 weeks, due to failure to achieve remission or due to relapse. According to this definition, 29 patients had a poor treatment outcome at week 4. The remaining 42 had a good treatment outcome at week 4, but 18 of them had relapsed by week 52.

All 71 patients received oral GC therapy. Those 71 patients had not received any immunosuppressive drugs or biologic agents at week 0. The initial mean dose ± S.D. of prednisolone was 0.82 ± 0.23 mg/kg/day, and 34 (47.3%) patients received GC pulse therapy at week 0. Clinical signs and symptoms resolved within 4 weeks in 42 (59%) of the patients, but the remaining 29 had a poor treatment outcome at week 4 despite initiation of GC therapy (Fig. 1). Fever (>39.0^o^C) was reported in 16 (55.2%) of these 29 patients at the time of treatment intensification by the second round of GC pulse therapy or any immunosuppressive drugs, elevated liver enzymes in 17 (58.6%), pericarditis in 3 (10.3%), pleuritis in 5 (17.2%), arthritis in 11 (37.9%), and HPS in 4 (13.8%) (Table 2). A second round of GC pulse therapy at the event of poor treatment outcome was added in 17 (58.6%) of the 29 poorly responding patients, and immunosuppressive drugs were added in 24 (82.8 %) of the 29 patients (Table 3). After the second round of GC pulse therapy or the addition of immunosuppressive drugs, 27 of the 29 patients achieved remission, and one patient died due to rapidly progressive HPS at week 5. One patient died because of severe bacterial infection at week 6. Patients who achieved remission within 4 weeks did not receive additional treatment intensification by any other immunosuppressive drugs, biologicals, or a second round of GC pulse therapy.

**Table 2:** Clinical signs and symptoms at the time of treatment intensification during 0–4 weeks and during 4–52 weeks

Clinical signs and symptoms	During 0–4 weeks *n*=29	During 4–52 weeks *n*=18
Fever (>39.0°C), %	55.2	52.9
Rashes, %	37.9	35.2
Lymphadenopathy, %	10.3	11.8
Hepatosplenomegaly, %	6.9	11.8
Elevated liver enzymes, %	58.6	29.4
Pericarditis, %	10.3	0
Pleuritis, %	17.2	0
Interstitial pneumonia, %	6.9	0
Arthritis, %	37.9	33.3
Hemophagocytic syndrome, %	13.8	0
DIC, %	13.8	0
CRP elevation without clinical signs and symptoms, %	0	11.1

*DIC* disseminated intravascular coagulation, *CRP* C-reactive protein

**Table 3:** Treatment regimen in the patients with and without the event of poor treatment outcome

Treatment	With the event^a^ (*n*=29)	Without the event (*n*=42)	*p*-value
Initial PSL dose (mg/kg/day) at week 0, mean ± SD	0.97 ± 0.15	0.72 ± 0.23	<0.001
GC pulse at week 0, *n* (%)	23 (79.3)	11 (26.2)	<0.001
Additional GC pulse within 4 weeks, *n* (%)	17 (58.6)	0	-
Additional immunosuppressive drugs at the event of poor treatment outcome, *n* (%)	24 (82.8)	0	-
MTX, *n* (%)	10 (34.5)	-	-
CyA, *n* (%)	4 (13.8)	-	-
Tac, *n* (%)	4 (13.8)	-	-
TCZ, *n* (%)	2 (6.9)	-	-
MTX + TCZ, *n* (%)	1 (3.4)	-	-
CyA + TCZ	3 (10.3)	-	-

*GC* glucocorticoid, *MTX* methotrexate, *CyA* cyclosporine, *Tac* tacrolimus, *TCZ* tocilizumab

^a^Event of a poor treatment outcome, which was defined as failure to achieve remission or relapse after achieving remission within 4 weeks, followed by administration of two or more rounds of GC pulse therapy or of any other immunosuppressive drugs

### Relapse after achieving clinical remission at week 4

Overall, 42 patients achieved remission by 4 weeks on oral GC either with or without a single GC pulse. Relapse after achieving clinical remission was observed in 18 of these patients during a 4–52-week follow-up (Fig. 1). The median time to relapse was 21 weeks (interquartile range 16–27). Clinical signs and symptoms at the time of relapse are shown in Table 2. Interestingly, unlike patients with a poor treatment outcome during 0–4 weeks, no severe organ damage was observed at the time of relapse between 4 and 52 weeks.

### Clinical characteristics of patients with a poor treatment outcome at week 4

Patients with poor treatment outcomes had more serositis and HPS at baseline, and a significantly higher mSFS. WBC count, NLR, serum ferritin, AST, and LDH levels at baseline were higher in patients with a poor treatment outcome, and the modified Pouchot score and severity index were also higher (Table 1). The initial prednisolone dose was higher and initial GC pulse treatment was more frequent for patients with a poor treatment outcome than in the remaining patients (Table 3).

In the sub-analysis for patients with and without GC pulse therapy at week 0, WBC count, NLR, and the modified Pouchot score at baseline were higher in the patients with the poor treatment outcome who received GC pulse therapy at week 0 than in those without the poor treatment outcome, while these were not observed in patients without GC pulse therapy at week 0 (Table S1).

### Factors associated with poor treatment outcome

We selected WBC count, Plt count, AST, LDH, and ferritin as factors potentially associated with poor treatment outcome at week 4 because these parameters are useful for the early diagnosis of MAS [16] or HPS [17]. The mSFS was included in the model as a measure of the severity of organ damage due to AOSD. Univariable logistic regression analysis revealed that WBC, NLR, ferritin, and mSFS were indeed significantly associated with poor treatment outcome at week 4. We then conducted multivariable logistic regression analyses (model 1), selecting age, WBC count, Plt count, ferritin, LDH, and mSFS as covariates of interest (Table 4). Notably, only WBC count remained significantly associated with poor outcome (odds ratio per 1000/μl increment: 1.12, 95% confidential interval [CI] 1.04–1.29), while Plt count, ferritin, LDH, and mSFS were no longer statistically significant (Table 4). We also confirmed that the correlation coefficient between WBC count and LDH was *r* = 0.120 (*p* = 0.330), WBC and ferritin was *r* = 0.306 (*p* = 0.01), WBC count and CRP was *r* = 0.648 (*p* < 0.001), and WBC count and mSFS was *r* = 0.156 (*p* = 0.201).

**Table 4:** Associated factors with the poor treatment outcome at week 4

	Univariable analysis		Multivariable analysis (model 1)^a^		Multivariable analysis (model 2)^b^	
	OR (95% CI)	*p*	OR (95% CI)	*p*	OR (95% CI)	*p*
Age, per 1 year increment	1.02 (0.99–1.04)	0.259	1.00 (0.97–1.04)	0.995	1.00 (0.97–1.04)	0.839
WBC, per 1000/μl increment	1.14 (1.05–1.24)	0.003	1.16 (1.04–1.29)	0.010		
NLR	1.07 (1.02–1.12)	0.006			1.06 (1.00–1.13)	0.063
Plt, per 1×10^4^/μl increment	0.97 (0.94–1.01)	0.180	0.98 (0.92–1.04)	0.457	1.01 (0.96–1.06)	0.659
Ferritin, per 1000ng/ml increment	1.05 (1.01–1.10)	0.014	1.02 (0.98–1.06)	0.450	1.01 (0.96–1.06)	0.712
LDH, per 100U/l increment	1.05 (0.98–1.12)	0.147	1.19 (0.95–1.49)	0.125	1.36 (1.02–1.81)	0.039
AST	1.00 (1.00–1.00)	0.829				
mSFS	2.25 (1.18–4.29)	0.014	1.64 (0.79–3.42)	0.186	1.17 (0.50–2.78)	0.718
Modified Pouchot score	2.00 (1.37–2.90)	<0.001				
Hemophagocytic syndrome	4.14 (0.97–17.6)	0.055				
Severity Index	1.89 (1.27–2.81)	0.002				

*WBC* white blood cell count, *NLR* neutrophil-lymphocyte ratio, *Plt* platelet count, *LDH* lactate dehydrogenase, *AST* aspartate transferase, *mSFS* modified systemic feature score, *Severity index* severity index of Japanese Ministry of Health, Labour and Welfare

^a^Age, WBC, Plt, ferritin, LDH, and mSFS were selected as covariates of interest. Modified Pouchot score and severity index were not included in the multivariable model because these contained WBC and ferritin

^b^Age, NLR, Plt, ferritin, LDH, and mSFS were selected as covariates of interest

The correlation coefficient between WBC count and NLR was *r* = 0.623 (*p* <0.001), and NLR was included in the multivariable analysis (model 2) instead of the WBC count. NLR also numerically increased the risk of poor treatment outcome at week 4, but it was not statistically significant (Table 4).

### The WBC count at baseline predicts poor treatment outcome at week 4

**Fig. 2:**
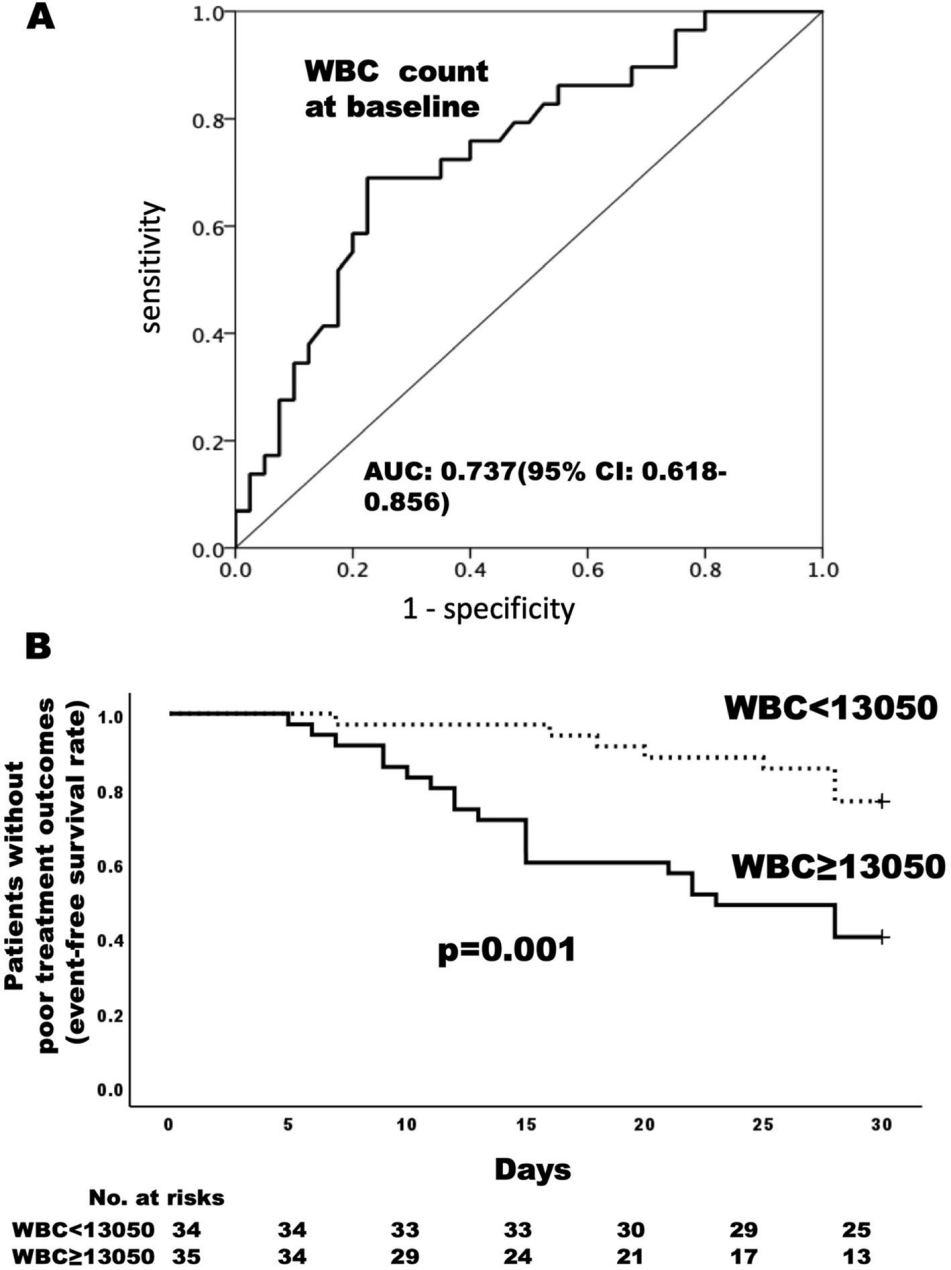
Association of WBC count with a poor treatment outcome during 4 weeks. Receiver-operating characteristic (ROC) curve for WBC (**A**). The area under the ROC curve was 0.737 (95% CI: 0.618-0.856, p=0.001); the analysis showed that the best cut-off for WBC was 13,050/μl, providing a sensitivity of 72% and a specificity of 65%. Analysis using the Kaplan-Meier method and the log-rank test (**B**). Cumulative rate of patients with a poor treatment outcome was 60.0% in those with WBC ≥13,050/μl and 23.5% in those with WBC <13,050/μl

We performed a ROC curve analysis in order to investigate the power of the WBC count for predicting the likelihood of a poor treatment outcome at week 4 (Fig. 2A). The area under the ROC curve was 0.737 (95 %CI: 0.618–0.856, *p*=0.001) and analysis of the AUC showed that the best cut-off for the WBC count was 13,050/μl for predicting a poor treatment response, with a sensitivity of 72% and specificity of 65%. The cumulative proportion of patients with a poor treatment outcome over 4 weeks was 60.0% in those with a WBC ≥13,050/μl but only 23.5% with a WBC <13,050/μl. The mean time to the event was significantly shorter by log-rank testing for patients with a WBC ≥13,050/μl relative to those with a WBC <13,050/μl (21.3 ± 1.5 vs. 27.9 ± 0.9 days) (Fig. 2B).

In sub-analysis for female patients and young adults (under 40 years of age), the mean time to the event was significantly shorter by log-rank testing for the patients with a WBC ≥13,050/μl relative to those with a WBC <13,050/μl (female patients: 21.5 ± 1.1 vs. 27.9 ± 1.0 days; young adults: 20.8 ± 2.9 vs. 29.8 ± 0.2 days) (Figure S1A and 1B).

### Safety

Adverse events over the 52-week follow-up in all 71 patients were evaluated. In the 29 patients with a poor treatment outcome, serious bacterial infections occurred in 3 (10.3%), cytomegalovirus infection in 7 (24.1%), deep fungal infection in 1 (3.4%), and bone fracture in one. Two of the 29 (6.9%) patients died due to severe bacterial infections at weeks 6 and 18. In 42 patients without such a poor treatment outcome, serious bacterial infection occurred in only 1 (2.4%), cytomegalovirus infection in 2 (4.8%), and deep fungal infection in one (2.4%). Bacterial pneumonia, pneumocystis pneumonia, herpes zoster, tuberculosis, and non-tuberculous mycobacterial infections were not reported.

## Discussion

The present study found that about 40% of newly diagnosed patients with AOSD had a poor response to initial GC therapy within the first 4 weeks. We assessed mSFS and HPS as indicators of the severity of organ damage and confirmed that patients with a poor treatment outcome had higher mSFS and more HPS than those responding to treatment, as previously reported [18]. However, our multivariable analysis revealed that increased WBC count was independently associated with poor response to initial GC therapy, while the mSFS and HPS, as well as other candidate factors (thrombocytopenia, elevated liver enzymes, high serum ferritin), were not. Thus, the novel observation here is that increased baseline WBC count is an independent important prognostic factor predicting the response to initial GC therapy of newly diagnosed patients with AOSD.

AOSD is heterogeneous, with patients manifesting different proportions of systemic features and organ damage depending on the type of systemic or chronic articular pattern [19]. Nonetheless, in the present study, epidemiological findings and the proportions of patients with pleuritis, pericarditis, HPS, and DIC at AOSD onset were similar to those in previous cohort studies in Japan and other countries, and the relapse rate was almost the same as in these previous studies [20–26]. In our cohort, 23.9% of the 71 patients had arthritis at the onset, but no high-fever (≥39.0°C), serositis, ILD, HPS, or DIC. Interestingly, the present study (Table 2) and previous studies showed that patients suffered less serious complications at relapse than at initial onset [20].

The classification criteria were proposed for MAS of febrile systematic JIA [27], with 45% and 56% of AOSD patients meeting these criteria according to two published studies [28, 29]. Unfortunately, we were unable to examine the rate of MAS complications according to these sJIA classification criteria because data on fibrinogen and triglycerides were not available in many cases. In the present study, 14% of patients did have HPS diagnosed by bone marrow aspiration, but we could not establish who had clinically diagnosed MAS in the absence of HPS.

In patients with AOSD, neutrophils infiltrate into the skin, lymph nodes, and liver [2, 30], resulting in macrophage activation, and induction of pro-inflammatory cytokines [31–34]. Importantly, the present study suggests that increased WBC counts predict a poor prognosis of AOSD early on, before complications of HPS or DIC may set in. More intensive combination therapies with high-dose GC and other immunosuppressive drugs might therefore be an appropriate induction treatment strategy for patients with WBC ≥13,050/μl at baseline, before progression to MAS. Additionally, previous studies had suggested that serum IL-18 levels are useful for predicting MAS in s-JIA [16, 35, 36]. In the present study, the correlation coefficients between WBC count and serum pro-inflammatory cytokines were not studied. Further studies with a prospective and multicentered design in Rare Disease Data Registry of Japan would provide valuable data on the role of pro-inflammatory cytokines including IL-18 as well as high WBC counts also in AOSD (trial number: UMIN000043071).

Different prognostic factors have been reported for AOSD, depending on study design, the treatment outcomes selected as the end-point, and heterogeneity of AOSD [18, 20–24, 37–49]. A previous nationwide epidemiological survey of AOSD in Japan in 2010 showed that patients receiving GC pulse therapy were those who had had more serositis, HPS, higher neutrophil counts, and higher serum ferritin levels than those who did not receive GC pulse therapy [23]. Previous studies showed that HPS, lymphadenopathy, the presence of lung disease, and liver involvement were associated with a poor prognosis. A higher systemic score and elevated serum ferritin were also reported as negative factors [40, 41, 44, 45, 47]. The univariable analysis of the present study also confirmed that these factors were associated with poor treatment outcome, and notably our multivariable analysis confirmed the clinical significance of increased WBC count as an independent prognostic factor. In the stratification analysis of the present study, the increased WBC count was also a poor prognostic factor in female patients or young-adult patients. However, it was not likely to be a prognostic factor in patients who did not receive GC pulse therapy at baseline. Since we were not able to validate our results by other registries, stratified analysis considering severity and disease type should be conducted in a multicenter prospective cohort study with a larger number of cases.

When interpreting the results of our study, the inherent limitations of a monocentric observational retrospective study must be considered. First, our cohort may have included more patients with a severe course of systemic AOSD, because it consisted only of patients referred from other institutes who required specialized treatment at our university medical center. Second, the attending physician might have initiated intensive treatment for patients with severe organ damage or HPS at baseline, regardless of treatment responsiveness. However, we confirmed that additional treatment intensification was not conducted for the purpose of reduction of GC dose. Combination therapy of GCs and immunosuppressive drugs was not administered early in the remission induction therapy. Third, we were unable to analyze factors associated with poor treatment outcome at 1 year, because 14 patients were transferred to other hospitals after achieving remission.

## Conclusions

The present study showed that about 40% of newly diagnosed AOSD patients had a poor response to the initial standard oral GC therapy with or without GC pulse therapy and that the severity of AOSD (modified Pouchot score and severity index), HPS, and mSFS were associated with this poor treatment outcome. Multivariable analysis clearly demonstrated that a higher WBC count at baseline independently predicted poor treatment responsiveness and that the most valuable cutoff was a WBC count of ≥13,050/μl. Our findings provide important information for determining the initial treatment strategy of newly diagnosed AOSD. Prospective cohort studies need to be conducted to validate this result.

## Supplementary Information

**Additional file 1: Figure S1.** Association of WBC count with a poor treatment outcome during 4 weeks in females (A) or young adults (B).

**Additional file 2: Table S1.** Clinical characteristics of patients at onset with or without glucocorticoid pulse therapy.

## Data Availability

All of the data supporting the conclusions of this article are included within the article.

## Abbreviations

ALT: Alanine aminotransferase
AOSD: Adult-onset Still’s disease
AST: Aspartate aminotransferase
CI: Confidential interval
CRP: C-reactive protein
CyA: Cyclosporine
DIC: Disseminated intravascular coagulation
ESR: Erythrocyte sedimentation rate
GC: Glucocorticoid
Hb: Hemoglobin
HPS: Hemophagocytic syndrome
LDH: Lactate dehydrogenase
MAS: Macrophage activation syndrome
mSFS: Modified systemic feature score
MTX: Methotrexate
NLR: Neutrophil-lymphocyte ratio
Plt: Platelet
PSL: Prednisolone
sJIA: Systemic juvenile idiopathic arthritis
ROC: Receiver-operating characteristic
Severity Index: The severity index of the Japanese Ministry of Health, Labour and Welfare
S.D.: Standard deviation
Tac: Tacrolimus
TCZ: Tocilizumab
WBC: White blood cell
