# Supplementary material for: Associated factors with poor treatment response to initial glucocorticoid therapy in patients with adult-onset Still’s disease
Source: Arthritis Res Ther. 2022 Apr 29;24:92. doi: 10.1186/s13075-022-02780-3 (PMC9052454; doi:10.1186/s13075-022-02780-3)
Supplement: Supplementary file 1 — Additional file 1: Figure S1. Association of WBC count with a poor treatment outcome during 4 weeks in females (A) or young adults (B). [file 13075_2022_2780_MOESM1_ESM.docx]

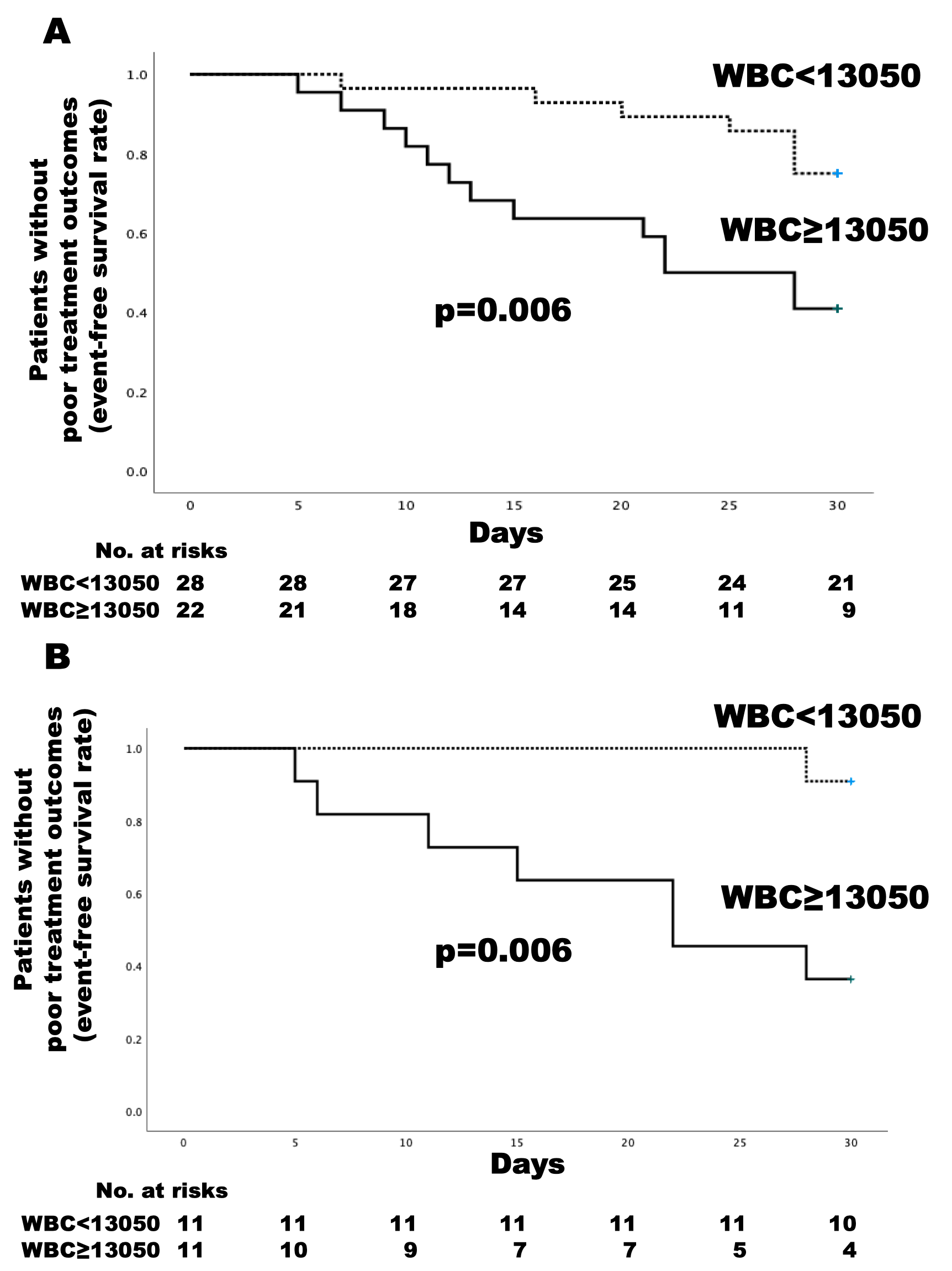


Supplementary Figure 1. Association of WBC count with a poor treatment outcome during 4 weeks in females (A) or young adults (B)
