# Supplementary material for: Associated factors with poor treatment response to initial glucocorticoid therapy in patients with adult-onset Still’s disease
Source: Arthritis Res Ther. 2022 Apr 29;24:92. doi: 10.1186/s13075-022-02780-3 (PMC9052454; doi:10.1186/s13075-022-02780-3)
Supplement: Supplementary file 2 — Additional file 2: Table S1. Clinical characteristics of patients at onset with or without glucocorticoid pulse therapy. [file 13075_2022_2780_MOESM2_ESM.docx]

Supplementary Table 1. Clinical characteristics of patients at onset with or without glucocorticoid pulse therapy

|  | With GC pulse therapy | | | | Without GC pulse therapy | | |
| --- | --- | --- | --- | --- | --- | --- | --- |
|  | With the  event^a^  (n = 23) | Without the  event  (n = 11) | *p*-value | With the  event^a^  (n = 6) | | Without the  event  (n = 31) | *p*-value |
| Age, years,  mean ± S.D. | 55.4  ± 19.0 | 45.6  ± 16.8 | 0.155 | 51.2 ±19.7 | | 51.0 ± 17.8 | 0.980 |
| Female patients, % | 69.6 | 63.6 | 0.511 | 66.7 | | 74.2 | 0.525 |
| Hemophagocytic syndrome, % | 30.4 | 18.2 | 0.375 | 0.0 | | 3.2 | 0.838 |
| DIC, % | 8.7 | 9.1 | 0.704 | 0.0 | | 3.2 | 0.838 |
| mSFS score,  mean ± S.D. | 3.65  ± 0.98 | 3.00  ± 0.89 | 0.072 | 3.33 ± 0.52 | | 3.03 ± 0.84 | 0.403 |
| WBC, /μl, mean ± S.D. | 18452  ± 7050 | 10476  ± 7444 | 0.005 | 11900  ± 4658 | | 12047  ± 5555 | 0.952 |
| Neutrophil, /μl,  mean ± S.D. | 16239  ± 6688 | 9079  ± 7256 | 0.008 | 9550  ± 4691 | | 9876  ± 5268 | 0.889 |
| Lymphocyte, /μl,  mean ± S.D. | 945  ± 772 | 830  ± 487 | 0.657 | 1317  ± 164 | | 1128  ± 538 | 0.138 |
| NLR, mean ± S.D. | 23.37  ± 13.45 | 13.22  ± 9.88 | 0.033 | 7.73  ± 5.07 | | 10.88  ± 8.41 | 0.388 |
| Modified Pouchot score, mean ± S.D. | 7.48  ± 1.70 | 5.91  ± 1.45 | 0.013 | 6.50  ± 1.05 | | 5.42  ± 1.61 | 0.125 |
| Severity index,  mean ± S.D. | 4.43  ± 1.70 | 3.36  ± 1.96 | 0.112 | 3.00  ± 0.63 | | 2.55 ± 1.03 | 0.183 |

^a^Event of a poor treatment outcome, which was defined as failure to achieve remission or relapse after achieving remission within 4 weeks, followed by administration of two or more rounds of GC pulse therapy or of any other immunosuppressive drugs.

GC, glucocorticoid; S.D., standard deviation; WBC, white blood cell; DIC, disseminated intravascular coagulation; mSFS, modified systemic feature score; NLR, neutrophil-lymphocyte ratio; Severity index; severity index of Japanese Ministry of Health, Labour and Welfare.
